# *Pseudosuccinea columella*: age resistance to *Calicophoron daubneyi* infection in two snail populations

**DOI:** 10.1051/parasite/2015003

**Published:** 2015-02-10

**Authors:** Yasser Dar, Daniel Rondelaud, Philippe Vignoles, Gilles Dreyfuss

**Affiliations:** 1 Department of Zoology, Faculty of Science, University of Tanta Tanta Egypt; 2 INSERM 1094, Faculties of Medicine and Pharmacy 87025 Limoges France

**Keywords:** *Calicophoron daubneyi*, Cercaria, Experimental infections, Prevalence, *Pseudosuccinea columella*, Rediae

## Abstract

Individual infections of Egyptian and French *Pseudosuccinea columella* with five miracidia of *Calicophoron daubneyi* were carried out to determine whether this lymnaeid was capable of sustaining larval development of this parasite. On day 42 post-exposure (at 23 °C), infected snails were only noted in groups of individuals measuring 1 or 2 mm in height at miracidial exposure. Snail survival in the 2-mm groups was significantly higher than that noted in the 1-mm snails, whatever the geographic origin of snail population. In contrast, prevalence of *C. daubneyi* infection was significantly greater in the 1-mm groups (15–20% versus 3.4–4.0% in the 2-mm snails). Low values were noted for the mean shell growth of infected snails at their death (3.1–4.0 mm) and the mean number of cercariae (<9 in the 1-mm groups, <19 in the 2-mm snails). No significant differences between snail populations and snails groups were noted for these last two parameters. Most infected snails died after a single cercarial shedding wave. Both populations of *P. columella* showed an age resistance to *C. daubneyi* infection and only juveniles measuring 2 mm or less in shell height at exposure can ensure larval development of this digenean up to cercarial shedding.

## Abstract (fr)

Des infestations individuelles de *Pseudosuccinea columella* provenant d’Egypte et de France, avec cinq miracidiums de *Calicophoron daubneyi*, ont été réalisées afin de déterminer si cette limnée était capable de soutenir le développement larvaire de ce parasite. Au 42^ème^ jour post-exposition (à 23 °C), seuls les groupes constitués par des individus mesurant 1 ou 2 mm lors de l’exposition miracidienne ont montré la présence de mollusques infestés. La survie des limnées dans le groupe 2 mm est significativement meilleure que celle trouvée chez les individus de 1 mm, quelle que soit l’origine géographique de la population. Par contre, la prévalence de l’infestation avec *C. daubneyi* est significativement plus importante dans les groupes 1 mm (15–20 % au lieu de 3,4–4,0 % chez les individus de 2 mm). De faibles valeurs ont été notées pour la croissance moyenne des individus infestés à leur mort (3.1-4.0 mm) et le nombre moyen de cercaires émises (< 9 dans les groupes 1 mm, < 19 chez les individus de 2 mm). Aucune différence significative entre les deux populations et également entre les groupes n’a été trouvée pour ces deux derniers paramètres. La plupart des individus infestés sont morts après une seule vague d’émission cercarienne. Les deux populations de *P. columella* montrent une résistance à l’infestation par *C. daubneyi* en fonction de l’âge des individus et seuls les juvéniles mesurant 2 mm de hauteur ou moins lors de l’exposition peuvent assurer le développement larvaire de ce Digène jusqu’aux émissions cercariennes.

## Introduction

Paramphistomosis due to *Calicophoron daubneyi* Dinnik, 1962 [15] affects numerous cattle, sheep, and goat herds in most countries of Western Europe. This parasite may cause significant problems because of its immature stages which migrate within the small intestine of these definitive hosts [23]. In France, the prevalence of *C. daubneyi* infection in cattle living in the Limousin region increased from the 1990s up to 44.7% in 1999 [26, 40] and this rate has still remained high since 2000 in spite of repeated anthelmintic treatments administered to ruminants [18]. In this country, the snail *Galba truncatula* O.F. Müller, 1774 [27] is known to be the main intermediate host of this parasite [33]. Another lymnaeid, *Lymnaea glabra* O.F. Müller, 1774 [27], was also reported as a natural snail host for this digenean. This species cannot sustain complete larval development of *C. daubneyi* [1]. However, the coinfection of juvenile and pre-adult *L. glabra* with *C. daubneyi* and *Fasciola hepatica* Linnaeus, 1758 [24] (both digeneans often infected the same cattle in central France [36]) resulted in the development of *F. hepatica*, *C. daubneyi,* or both in these snails [2]. This last finding was verified in the field by detecting numerous *L. glabra* harboring larval forms of either digenean [3–5]. In contrast, the infection of older *L. glabra* (>6 mm in shell height) with the same miracidial sequence was always negative, thus demonstrating the existence of age resistance of these snails to coinfection with *C. daubneyi* and *F. hepatica* [1].

As the distribution of freshwater gastropod species in an ecosystem can change over time, local malacofauna may be enriched with the introduction of invasive species such as *Pseudosuccinea columella* Say, 1817 [37]. This lymnaeid of North American origin has successfully been introduced into Africa, Europe, Oceania, and South America [12, 41]. Its shell height at the adult stage may reach 15–18 mm [10, 21]. As the presence of this lymnaeid in France has been reported by Pointier et al. [31], it was interesting to determine whether this wild population found in the Lot department might sustain larval development of local digeneans such as *F. hepatica* and/or *C. daubneyi*. This snail was already known to be widely susceptible to *F. hepatica* infection, with prevalence of experimental infections varying from 10% to 100% [13, 20, 43]. In contrast, its role as an intermediate host in the life cycle of *C. daubneyi* was still not known. As *F. hepatica* and *C. daubneyi* were often found in the same French cattle [40] and could infect the same snail host (*G. truncatula*), the aim of this paper was to determine whether *P. columella* might play a role as an intermediate host in *C. daubneyi* transmission. To verify this possibility, experimental infections of juvenile and pre-adult *P. columella* (1–6 mm in shell height at miracidial exposure) with *C. daubneyi* were carried out under laboratory conditions using two snail populations (Egypt, France) and several French isolates of cattle-derived miracidia.

## Materials and methods

### Snails and parasite

The first population of *P. columella* used for the experiments was one from Egypt and was living in a water body (29°20′2.77ʺ N, 31°12′17.83ʺ E) at Al-Wasta, governorate of Beni Suef. The other was found in two sites (44°23′27.31ʺ N, 0°32′2.43ʺ E and 44°23′31.18ʺ N, 0°29′59.30ʺ E) located near Castelmoron along the banks of the Lot River, department of Lot, south-western France. Adult snails, measuring 10–15 mm in height, were collected in March 2013 from the first populations and in September to October 2013 from the other. They were transported to the laboratory and placed in 10-L covered aquaria with five snails per liter of permanently-oxygenated spring water. These aquaria were maintained at constant laboratory conditions: temperature, 23° ± 1 °C; light/dark period, 12 h/12 h. Dissolved calcium concentration in spring water was 35 mg/L. Snails fed on pesticide-free fresh lettuce leaves *ad libitum* and spring water in aquaria was changed weekly. Egg masses laid by these adult snails were collected and placed into small rearing aquaria. Newly hatched snails fed on finely powdered lettuce and those, which attained 1 mm (24 h of life), 2 ± 0.1 mm (5 days of life), 3 ± 0.1 mm, 4 ± 0.1 mm, 5 ± 0.1 mm, or 6 ± 0.1 mm in shell height, were used. For each *P. columella* population, a total of 800 snails were subjected to experimental infections.

To obtain *C. daubneyi* eggs, adult worms were collected from the rumen of infected cattle at the Limoges slaughterhouse (Central France) and dipped in a physiological saline solution (0.9% NaCl, 0.45% glucose) before being placed at 37 °C for 3 h. These eggs were washed several times with spring water and immediately incubated in the dark at 20 °C for 20 days [29].

### Experimental protocol

Two experiments were carried out in the present study. The aim of the first was to determine the aptitude of *P. columella* as a snail host for *C. daubneyi.* Six groups were constituted for each population (Table 1), with 100 individuals in the 1-mm group, 100 in the 2-mm group, and 50 in each of the other four. Each snail was subjected to five miracidia of *C. daubneyi* for four hours at 23 °C in 3.5 mL spring water. The choice of this sequence for infecting these 12 groups came from the report by Dar et al. [14] on *P. columella*. Snails were then raised for 42 days in individual 50-mm Petri dishes (10 mL spring water per recipient). In each dish, a piece of pesticide-free fresh lettuce leaf was placed. Petri dishes were then put in the same air-conditioned room at 23° ± 1 °C as parent snails. Daily surveillance was carried out to change spring water and food if necessary. On day 42 post-exposure (p.e.), surviving snails were dissected under a stereomicroscope to detect the presence of larval forms of *C. daubneyi* within their bodies and count free rediae and free cercariae.

**Table 1.** Snail survival on day 30 post-exposure, prevalence of *Calicophoron daubneyi* infection, and numbers of free rediae and free cercariae in 12 groups of *Pseudosuccinea columella* subjected to individual quinque-miracidial exposures and dissected on day 42. Fifty snails of each group were exposed on day 1, except for 100 snails in each 1-mm and 100 in each 2-mm group.

Snail population and group	Number of surviving snails (%)	Number of infected snails (prevalence %)	Number of larval forms: mean ± *SD*	
			Free rediae	Free cercariae
Egypt (mm)				
1	42 (42.0)	9 (21.4)	2.7 ± 1.1	5.3 ± 2.5
2	85 (85.0)	3 (3.5)	3.3 ± 1.4	8.4 ± 4.1
3	47 (94.0)	0 (–)	–	–
4	49 (98.0)	0 (–)	–	–
5	48 (96.0)	0 (–)	–	–
6	49 (98.0)	0 (–)	–	–
France (mm)				
1	37 (37.0)	6 (16.2)	3.4 ± 1.7	4.7 ± 3.5
2	79 (79.0)	1 (1.2)	4	7
3	45 (90.0)	0 (–)	–	–
4	47 (94.0)	0 (–)	–	–
5	47 (94.0)	0 (–)	–	–
6	49 (98.0)	0 (–)	–	–

As free cercariae were only noted in the 1- and 2-mm groups from each population (Table 1), a second experiment was carried out to determine the characteristics of these *C. daubneyi* infections in snails belonging to the above groups and follow the dynamics of their cercarial shedding. Two hundred 1-mm and two hundred 2-mm snails were used for each population (Table 2). Snail exposure to miracidia and maintenance were similar to those in the first experiment. Spring water and food were changed, if necessary, every day until snail death. When the first cercarial shedding occurred, surviving snails were subjected to a thermal shock every three days by placing their Petri dishes at 10°–13 °C for 3 h to stimulate cercarial exit [36, 44]. After their emergence, cercariae were counted and removed from Petri dishes. At the death of each infected snail, its shell was measured using callipers.

**Table 2.** Snail survival on day 30 post-exposure, prevalence of snail infection, shell growth of infected snails, and number of shed cercariae in four groups of *Pseudosuccinea columella* exposed to *Calicophoron daubneyi.* Two hundred snails of each group were exposed on day 1.

Snail population and group	Number of surviving snails (%)	Number of infected snails (prevalence %)	Shell growth (mm) of infected snails*	Number of shed cercariae*
Egypt (mm)				
1	94 (47.0)	19 (20.2)	2.7 ± 1.0	8.1 ± 3.1
2	175 (87.5)	7 (4.0)	3.4 ± 1.5	14.3 ± 5.3
France (mm)				
1	67 (33.5)	9 (13.4)	3.1 ± 0.8	6.5 ± 3.6
2	148 (74.0)	4 (2.7)	4.0 ± 1.8	16.3 ± 5.9

* Mean value ± *SD*.

### Data analysis

In both experiments, the first two parameters were snail survival on day 30 p.e. and the prevalence of *C. daubneyi* infection calculated in relation to the number of snails surviving on day 30 p.e. For each parameter, the differences were analyzed using a χ^2^ test.

Two other parameters in the first experiment were the number of free rediae and that of free cercariae counted in dissected snails. In the second experiment, the shell growth of infected snails at their death, the total number of shed cercariae, and the number of shedding waves for each infected snail [30] were also considered. Individual values recorded for these last five parameters were averaged and standard deviations were established for each snail group. Normality of these values was analyzed using the Shapiro-Wilk test [39]. According to results given by this test, one-way analysis of variance (ANOVA) or the Kruskal-Wallis test was used to establish levels of significance.

In the first experiment, the differences between survival rates, prevalence of infection and the numbers of larval forms were analyzed for each snail population considered separately. In the second experiment, the influence of snail population on the characteristics of *C. daubneyi* infection was evaluated by comparing differences between the five parameters for each snail group (1 or 2 mm). All statistical analyses were performed using Statview 5.0 software (SAS Institute Inc., Cary, NC, USA).

## Results

### Aptitude of *P. columella* as a snail host for *C. daubneyi* (experiment 1)

In both snail populations (Table 1), the survival rate on day 30 p.e. significantly increased (Egypt: χ^2^ = 61.28, *p* < 0.001; France: χ^2^ = 56.33, *p* < 0.001) from the 1-mm to the 3-mm groups, while the values did not significantly differ from each other in the upper size classes. Infected snails were only noted in the 1- and 2-mm groups, with prevalence of infection significantly higher (Egypt: χ^2^ = 10.52, *p* < 0.01; France: χ^2^ = 9.93, *p* < 0.01) in the 1-mm snails than in the others. After the dissection of infected snails, a mean of 2.7–3.3 and 3.4–4 free rediae were counted in the Egyptian and French snails, respectively, and no significant difference was noted for each population considered separately. The same finding was also noted for free cercariae, with a mean of 5.3–8.4 cercariae in Egyptian snails and 4.7–7 in the French snails.

### Characteristics of *C. daubneyi* infection in the two snail populations (experiment 2)

Table 2 gives the results of the second experiment. Compared to Egyptian snails, the survival rates in the French population were significantly lower (1-mm groups: χ^2^ = 7.58, *p* < 0.01; 2-mm snails: χ^2^ = 11.72, *p* < 0.001). Differences between prevalences were insignificant, whatever the snail group. The values noted for the mean shell growths of infected snails ranged from 3.1 to 4.0 mm and no significant difference was noted. Although there were slightly higher numbers of cercariae in the 2-mm groups than in the 1-mm snails (a mean of 14.3 cercariae versus 8.1 in the Egyptian groups, for example), the differences between these last values were also not significant.

In the Egyptian population, 13 snails (out of 19) and 6 (out of 7) in the 1- and 2-mm groups died after a single shedding wave of 1 or 2 days, while the other snails released their cercariae during two waves (data not shown). In the French population, the respective numbers of snails shedding their cercariae during a single wave were 8 (out of 9) and 4 (out of 4) in the 1- and 2-mm groups, respectively (data not shown). No significant difference between both populations was noted, whatever the snail group.

## Discussion

The present study demonstrates that a few juvenile *P. columella* (≤2 mm in shell height at miracidial exposure) are able to sustain complete larval development of *C. daubneyi* with cercarial shedding, while experimental infections in the upper size classes are negative. These results are difficult to comment on for the following three reasons: (i) although European cattle had regularly been imported into this country for many years [25], the existence of *C. daubneyi* in Egypt had not yet been reported in the literature; (ii) the presence of this digenean in the two French snail habitats seems improbable because these sites were away from cattle- or sheep-grazed meadows, and (iii) conflicting results were noted in the reports of authors who have carried out experimental infections of juvenile lymnaeids other than *G. truncatula* with *C. daubneyi*; Dinnik [15], Vassilev and Samnaliev [42], and Postal [32] did not succeed in their experimental infections. Sey [38] reported 3–5% prevalence in young *Lymnaea peregra* O.F. Müller, 1774 [27] (<1 month in age at exposure) infected with five miracidia per snail. Abrous [1] and Abrous et al. [4] have found live sporocysts and immature rediae in juvenile *Lymnaea fuscus* C. Pfeiffer, 1821 [30], *L. glabra*, *L. palustris* O.F. Müller, 1774 [27], and *L. stagnalis* Linnaeus, 1758 [24] (1–1.5 mm in shell height at exposure) after their infection with five miracidia per snail and their dissection on day 30 p.e. Two reliable hypotheses may be proposed to explain successful infections of juvenile *P. columella* with *C. daubneyi*. The first is to consider this result as a snail species-specific characteristic. However, a decrease in *P. columella* resistance to *C. daubneyi* infection, due to the conditions of snail breeding in the laboratory, cannot be completely excluded.

Owing to conflicting results (see above) reported by previous authors on experimental infections of juvenile lymnaeids with *C. daubneyi*, the results found in the present study were compared with those reported by Kendall [22], Berghen [7], Boray [8, 9], Busson et al. [11], Dreyfuss et al. [19], or Novobilský et al. [28] for infections of juvenile lymnaeids other than *G. truncatula* with *F. hepatica*. According to these authors, the survival and prevalence of infection of parasite-exposed newborns were low, while shed cercariae generally did not exceed 20 in number and are released during a single shedding wave. Moreover, the quantity of free rediae developing in these juveniles was also low, with only a few units per snail. When the shell height of snails increased at exposure, the survival rate and the number of shed cercariae increased, while prevalence of parasite infections decreased. As most findings reported by the above authors correspond with those reported in the present study, the conclusion adopted by Boray [9] can be proposed to comment on the results noted in *C. daubneyi*-infected juveniles of *P. columella*. In this lymnaeid species, a resistance to parasite infection would develop in the days which follow hatching of newborns and the size of 2 mm at miracidial exposure constitutes the onset of age resistance to *C. daubneyi*. Several hypotheses, such as the immaturity of the defence system in newborns and 1-mm young snails [17], the suppressive effect of trematode infection on snail’s immune system [16], or the type of nutrition and general fitness of juvenile snails [6], have been proposed to explain larval development of a digenean infection in juvenile lymnaeids.

In spite of the age resistance of *P. columella* to *C. daubneyi* infection, this situation may not be stable over the following years and a change in the susceptibility of these populations to this digenean can be expected if miracidia are permanently present in the habitats where snails are living. An argument supporting this last assumption came from the two reports by Rondelaud et al. [34, 35]. Pre-adults (4 mm in height at exposure) of three lymnaeid species (*L. fuscus*, *L. glabra*, *Radix balthica* Linnaeus, 1758 [24]) are known to be resistant to *F. hepatica* infection [9, 19, 28]. In contrast, the *F. hepatica* infection of several successive generations of pre-adults, coming from parents already infected with this parasite, resulted in a progressive increase in prevalence and intensity of snail infection [34, 35]. If in the future, *P. columella* colonizes French hydrographical networks up to open drainage systems, this lymnaeid might play an active role in *C. daubneyi* transmission and supplant the local common snail host, *G. truncatula*.

In conclusion, both populations of *P. columella* showed age resistance to *C. daubneyi* infection and only juveniles measuring 2 mm or less in shell height at exposure can ensure larval development of this digenean up to cercarial shedding.
